# BANTAM-01: A Multicenter, Open-Label, First-in-Human Phase 1 Dose Escalation and Expansion Study of ^225^Ac-GPC3 (BAY 3547926) in Patients with Advanced Hepatocellular Carcinoma

**DOI:** 10.2967/jnumed.125.270814

**Published:** 2026-03

**Authors:** Jorrit Tjeertes, Miroslav Dostalek, Charles Glaus, Vinita Gupta, Daneng Li, Antje Nestler, Vasiliki Pelekanou, Joerg Pinkert, Ida Ratih, Eric Smith, Shyamal Subramanyam, Arndt Vogel, Katrina Walker, Andrea Wilson Woods, Stefan Zimmermann, Henrik Junger

**Affiliations:** 1 Bayer AG, Basel, Switzerland;; 2 Bayer Research and Innovation Center, Bayer US, Cambridge, Massachusetts;; 3 City of Hope Comprehensive Cancer Center, Duarte, California;; 4 Bayer AG, Berlin, Germany;; 5 Bayer PLC, Reading, Berkshire, United Kingdom;; 6 Bayer US, Whippany, New Jersey;; 7 Toronto General Hospital and Princess Margaret Cancer Center, Toronto, Ontario, Canada;; 8 Hannover Medical School, Hannover, Germany;; 9 Blue Faery, Birmingham, Alabama; and; 10 University Hospital of Regensburg, Regensburg, Germany

**Keywords:** hepatocellular carcinoma, GPC3, targeted α-therapy, ^225^Ac, BANTAM-01

## Abstract

Despite the increasing availability of systemic therapies to treat advanced hepatocellular carcinoma (HCC), the prognosis remains poor. There is therefore an unmet need for targeted therapies with a novel mode of action that are more tolerable and effective than current treatment options. Targeted α-therapies selectively deliver high-energy α-particle–emitting radionuclides to tumor-associated cell surface antigens, inducing difficult-to-repair DNA double-stranded breaks, while limiting damage to surrounding tissue. Elevated expression of the oncofetal cell surface heparan sulfate proteoglycan, glypican-3 (GPC3), is seen in more than 70% of HCCs and is a negative prognostic indicator. BAY 3547926 (^225^Ac-GPC3) is an ^225^Ac-labeled antibody–chelator conjugate (ACC) that delivers ^225^Ac directly to GPC3-expressing cancer cells and is a potential first-in-class targeted α-therapy in advanced HCC. **Methods:** This multicenter, open-label, nonrandomized first-in-human phase 1 dose escalation and expansion study aims to evaluate the safety, tolerability, pharmacokinetics, and antitumor activity of ^225^Ac-GPC3 ACC, with or without preinjection of the nonradioactive GPC3 ACC, in participants with advanced HCC. The study has 3 parts: dose escalation, dose expansion as monotherapy, and safety run-in and dose expansion in combination with standard of care. Key eligibility criteria include diagnosis of locally advanced or metastatic and/or unresectable HCC, with disease not amenable to, or progressive disease after, curative surgery and/or locoregional therapies. To ensure only participants who are good candidates to benefit from ^225^Ac-GPC3 ACC enter the study, participants will be prospectively screened to confirm GPC3 membrane expression in a tumor specimen. All participants will receive study treatment on day 1 of a 6-wk cycle; additionally, participants in part 3 will receive standard-of-care treatment at the usual frequency. Participants will receive up to 4 doses of the study intervention, unless withdrawn from the study on disease progression, unacceptable toxicity, or fulfillment of any other withdrawal criteria. Primary endpoints include assessment of the occurrence and severity of treatment-emergent adverse events, recommended dose, objective response rate, disease control rate, duration of response, and progression-free survival. **Conclusion:** Study recruitment commenced in March 2025, and approximately 148 participants will be enrolled. The study will provide important preliminary insights on the efficacy and safety of ^225^Ac-GPC3 ACC therapy.

## Visual Abstract

**Figure:**
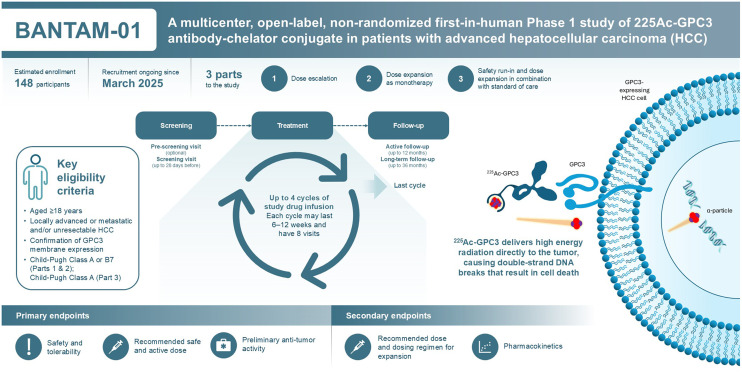

Primary liver cancer is the sixth most common cancer worldwide; there were almost 900,000 new diagnoses and over 750,000 deaths in 2022 (1,2), and the incidence is projected to reach 1.4 million cases by 2045 (3). Hepatocellular carcinoma (HCC), the most common form of liver cancer, accounts for approximately 75%–85% of cases (1). In the West, rates are climbing because of alcohol-related and metabolic liver disease, whereas in Asia, incidence is declining thanks to hepatitis B vaccination and antivirals for hepatitis B and C (4).

Patients with advanced HCC (Barcelona Clinic Liver Cancer stage C) are ineligible for curative treatments such as transplantation or resection but have access to systemic therapies (5,6). These include tyrosine kinase inhibitors such as sorafenib, lenvatinib, cabozantinib, and regorafenib, as well as antiangiogenic antibodies such as ramucirumab and bevacizumab (7). Standard of care (SoC) in the first-line setting is immune checkpoint inhibitor–based combination therapy, targeting either 2 checkpoints or combining checkpoint inhibitors with anti–vascular endothelial growth factor–directed agents, namely, atezolizumab/bevacizumab or durvalumab/tremelimumab. Tyrosine kinase inhibitors can be used when immune checkpoint inhibitors are unsuitable or as second-line options. Despite these advances, prognosis remains poor, with a 5-y survival rate of less than 20% (8,9). Responses with current immune checkpoint inhibitor–based therapies are observed in 20%–40% of patients, but up to nearly one third of tumors may be intrinsically resistant (10). There is therefore an unmet need for more effective and tolerable treatments with novel mechanisms of action.

Glypican-3 (GPC3) is an oncofetal cell surface heparan sulfate proteoglycan involved in various cancer-related signaling pathways (11,12). GPC3 is expressed during embryonic development; however, some expression is observed in normal and inflammatory tissues in adults, such as kidney, lung, and placenta (11). Elevated GPC3 membrane expression is seen in more than 70% of HCCs (13) and to a lower extent in other tumor types including squamous cell lung cancer, testicular germ cell tumors, and liposarcoma (14). Elevated GPC3 expression is a negative prognostic indicator in HCC (15), making it a promising therapeutic target.

Multiple GPC3-targeted therapies have been explored, including monoclonal antibodies, T-cell–engaging (TCE) antibodies, chimeric antigen receptor-T (CAR-T) cells, cancer vaccines, and antibody–drug conjugates (11). Codrituzumab, an anti-GPC3 monocolonal antibody, was tested as a monotherapy and with atezolizumab in phase 1 and 2 trials for advanced HCC, but the phase 2 trial ended early due to a lack of efficacy (16,17). TCE antibodies and GPC3-targeted CAR-T therapies, especially in combination with sorafenib or epidermal growth factor receptor–targeting therapies, have shown more promise (18,19). However, CAR-T therapies face challenges such as the need for lymphodepletion and the risk of cytokine release syndrome and neurotoxicity, limiting broader use. This underscores the need for new GPC3-targeted strategies.

Targeted α-therapies (TATs) deliver high-energy α-particle–emitting radionuclides directly to tumor-associated antigens, minimizing damage to adjacent nontumor tissue (20). Radionuclides such as ^225^Ac have high linear energy transfer and short range, causing clusters of hard-to-repair DNA double-strand breaks that trigger cell death (21). Several α-radionuclide–conjugated monoclonal antibodies have shown antitumor activity, supporting their further clinical development in cancer therapy (22–24).

The molecule BAY 3547926 (^225^Ac-GPC3) is an ^225^Ac-labeled antibody–chelator conjugate (ACC) in clinical development for the treatment of patients with advanced HCC. ^225^Ac-GPC3 ACC consists of the radioactive α-emitter ^225^Ac chelated to an anti-GPC3 monoclonal antibody, which delivers ^225^Ac directly to GPC3-expressing cancer cells (Fig. 1) and is a potential first-in-class TAT in advanced HCC. BANTAM-01 (**B**AY 3547926: **A N**ovel **T**herapeutic **^225^Ac M**odality) is a first-in-human study that aims to investigate ^225^Ac-GPC3 ACC safety, tolerability, pharmacokinetics, and antitumor activity (NCT06764316). Here, we present a comprehensive overview of the study design, including the patient selection strategy, clinical study protocol, and patient-centric approaches to recruit participants into the study.

**FIGURE 1.**
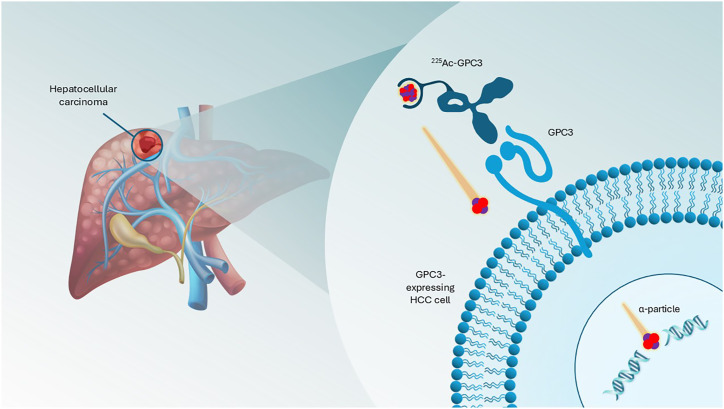
Mode of action of ^225^Ac-GPC3 ACC.

## PROTOCOL

### Study Design

The BANTAM-01 trial is a multicenter, open-label, nonrandomized first-in-human phase 1 dose escalation and expansion study evaluating the safety, tolerability, pharmacokinetics, and antitumor activity of ^225^Ac-GPC3 ACC therapy, with or without preinjection of a nonradioactive GPC3 ACC, alone or in combination with SoC in participants with advanced HCC. Nonradioactive GPC3 ACC is used as a preinjection before ^225^Ac-GPC3 ACC administration to ensure each participant receives the total mass dose of ^225^Ac-GPC3 ACC. This approach is based on preclinical and imaging data showing optimal biodistribution and tumor targeting at specific mass doses.

The study has 3 parts.

- **Part 1 (dose escalation):** evaluates the safety, tolerability, and recommended safe dose and regimen for dose expansion of ^225^Ac-GPC3 ACC, with or without preinjection of nonradioactive GPC3 ACC, given as monotherapy.
- **Part 2 (dose expansion as monotherapy):** aims to confirm the recommended dose and dosing regimen identified in part 1 by further characterizing the safety and tolerability and assessing the preliminary antitumor activity and pharmacokinetics of ^225^Ac-GPC3 ACC, given as monotherapy.
- **Part 3 (safety run-in and dose expansion in combination with SoC):** same as part 2, but with ^225^Ac-GPC3 ACC given in combination with SoC, currently atezolizumab (1,200 mg) or bevacizumab (15 mg/kg).

**FIGURE 2.**
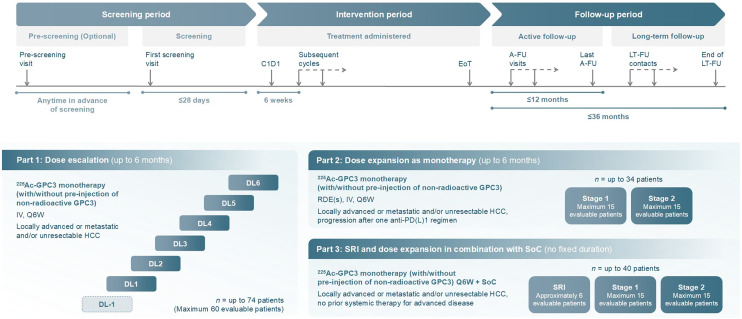
Study schema for BANTAM-01. In parts 2 and 3, early data review will be performed at end of stage 1, once all 15 participants have completed tumor assessments, to decide if study should continue to stage 2. A-FU = active follow-up; C1D1 = cycle 1 day 1; DL = dose level; EoT = end of treatment; IV = intravenous; LT-FU = long-term follow-up; Q3W = every 3 wk; Q6W = every 6 wk; RDE = recommended dose for expansion; SRI = safety run-in.

Recruitment began in March 2025 with the aim of enrolling around 148 participants in total to achieve approximately 126 evaluable participants. The study duration will be up to 49 mo in parts 1 and 2 (1 mo screening, up to 12-mo intervention period, and up to 36 mo follow-up). The intervention period in part 3 has no fixed duration, so the total study duration may be longer. The study schema is illustrated in Figure 2. Study endpoints are summarized in Table 1. The following institutional review boards and ethics committees have reviewed and approved this study: Surrey Borders Research Ethics Committee (United Kingdom), Research Ethics Committee of the Center hospitalier de l’Université de Montréal (Canada), Ontario Cancer Research Ethics Board (Canada), HM Hospitales Medical Research Ethics Committee (Spain), National Committee on Medical Research Ethics (Tukija) (Finland), and Comité d’Ethique de l’HUB (Hôpital Universitaires de Bruxelles) (Belgium).

**TABLE 1.** Study Endpoints

Primary endpoints	Secondary endpoints
Part 1: dose escalation	
Occurrence and severity of TEAEs Recommended dose based on occurrence of DLTs and preliminary antitumor activity (ORR*)	Recommended dosing based on occurrence and severity of TEAEs and DLTs, PK, immunogenicity, and preliminary antitumor activity (ORR*) ORR, DCR, DoR, and PFS* PK parameters including, but not limited to, *C*_max_ and AUC after single and multiple doses, where applicable
Part 2: dose expansion	
Occurrence and severity of TEAEs ORR, DCR, DoR, and PFS*	Recommended dosing based on safety, PK, immunogenicity and markers of pharmacodynamic activity, and efficacy assessments PK parameters including, but not limited to, *C*_max_ and AUC after single and multiple doses, where applicable
Part 3: dose expansion in combination with SoC	
Occurrence and severity of TEAEs ORR, DCR, DoR, and PFS*	Recommended dosing based on clinical data including, but not limited to, occurrence and severity of TEAEs and DLTs, PK and immunogenicity Recommended dosing based on severity of TEAEs, PK, immunogenicity PK parameters including, but not limited to, *C*_max_ and AUC after single and multiple doses, where applicable

* Using RECIST 1.1.

TEAE = treatment-emergent adverse event; DLT = dose-limiting toxicity; PK = pharmacokinetic; ORR = objective response rate; DCR = disease control rate; DoR = duration of response; PFS = progression-free survival; *C*_max_ = maximum concentration; AUC = area under the curve.

### Study Population

Participants aged 18 y or older with locally advanced or metastatic and/or unresectable HCC are eligible. Liver function status will be calculated during the screening period, and participants with Child-Pugh class A or class B7 are eligible for parts 1 and 2, whereas participants with Child-Pugh class A are eligible for part 3. In addition, participants must have adequate bone marrow and organ function. Patients with fibrolamellar HCC, sarcomatoid HCC, and mixed hepatocellular/cholangiocarcinoma subtypes will not be eligible. Full inclusion and exclusion criteria are listed in Supplemental Table 1 (supplemental materials are available at http://jnm.snmjournals.org).

Participants will be enrolled into the study after confirmation of GPC3 membrane expression in their tumor specimen. Participants will provide informed consent for the collection and testing of tumor tissue (archival/fresh) during the prescreening or screening period of the study. GPC3 expression will be tested by a College of American Pathologists/Clinical Laboratory Improvement Amendments accredited central laboratory using a test created and analytically validated according to 2017/746 in the European Union (25). Eligible participants are required to have GPC3 expression on their tumor samples above the technical limit of detection of the immunohistochemical test using the Roche BenchMark ULTRA system.

### Treatment

#### Administration

Administration of ^225^Ac-GPC3 ACC (and if applicable, nonradioactive GPC3 ACC) will be via controlled infusion. If nonradioactive GPC3 ACC is needed to achieve the required total dose of ^225^Ac-GPC3 ACC, it will be administered first.

All participants will receive study treatment on day 1 of a 6-wk cycle; additionally, participants in part 3 will receive SoC treatment at the usual frequency. If infusion-related or allergic reactions occur after the first dose on day 1 of each cycle, the infusion rate will be reduced or treatment paused or permanently discontinued.

The minimal dosing interval of 5 wk is set to optimize the initial dose intensity for efficacy while allowing adequate time for hematologic recovery, particularly concerning bone marrow function. Participants will receive up to 4 doses of ^225^Ac-GPC3 ACC to allow for sustained efficacious exposure while minimizing the risk for cumulative toxicity. Treatment with SoC in part 3 will continue beyond the 4 doses of ^225^Ac-GPC3 ACC and will be administered until unacceptable toxicity or loss of clinical benefit, as determined by the investigator after assessment of radiographic and biochemical data and clinical status.

#### Dose Delays and Modifications

Participants will receive study intervention until disease progression, unacceptable toxicity, or until any other withdrawal criteria are met (Supplemental Table 2). Changes to dosing frequency may be implemented during the study on the basis of emerging safety data.

#### Treatment Discontinuation

Reasons for treatment discontinuation include participant or investigator request, confirmed radiologic disease progression as per RECIST version 1.1, serious adverse reaction, delay of next treatment cycle for more than 6 wk because of treatment-related toxicity, development of a second primary malignancy, starting treatment with any other anticancer therapy or other prohibited medication (Supplemental Table 3), development of any intercurrent illness or situation which may affect clinical assessments, loss to follow-up, and death.

All participants will have an end-of-treatment visit at the end of the intervention period. This visit will occur at the end of the last cycle, approximately 6–12 wk after the scheduled last dose of study treatment in parts 1 and 2 or once the decision is made to discontinue treatment, whichever is later. For part 3, the end-of-treatment visit will take place on unacceptable toxicity or loss of clinical benefit, withdrawal of consent, or death, whichever occurs first.

#### Follow-Up

The active follow-up period will last for up to 12 mo after the end of treatment, with clinic visits approximately every 6 wk. All participants discontinuing from the study intervention period (except for death, loss to follow-up, or withdrawal of consent) will have safety assessments at active follow-up visits.

Participants who start subsequent anticancer treatment will end active follow-up and will continue with long-term follow-up. This will continue for up to 36 mo after the end of treatment, with information collected approximately every 12 wk.

### Assessments

#### Imaging

Tumor assessments during the intervention period will occur every 6 wk (±7 d) through week 36 in parts 1 and 2 and week 48 in part 3 and then every 12 wk (±14 d) thereafter until initial criteria for disease progression are met. Tumor assessment will be repeated at the end-of-treatment visit or safety follow-up visit.

Tumor response assessment will be determined per RECIST 1.1 and modified RECIST. Contrast-enhanced CT is the preferred and recommended imaging modality for tumor response assessment; however, contrast-enhanced MRI may be used in circumstances where CT is contraindicated or not available.

Participants can also participate in a γ-imaging and dosimetry substudy to measure the absorbed radiation dose delivered by ^225^Ac-GPC3 ACC to healthy organs and tumor lesions.

#### Safety Assessments

Safety will be assessed by monitoring adverse events (AEs), serious AEs, and AEs of special interest (Supplemental Table 4).

Cardiac, hepatic, renal, bone health, hematologic and blood chemistry parameters, vital signs, and any abnormal findings observed during the performance of physical examinations will also be assessed.

### Statistical Analyses

This is primarily an exploratory safety and tolerability study. For part 1, no formal statistical sample size calculation is performed, consistent with standard practice in first-in-human trials. For parts 2 and 3, sample sizes are determined using a Bayesian 2-stage design. Decision thresholds are set at posterior probabilities of at least 90% for efficacy (go) and at least 80% for early futility (no go). Sample sizes and critical responder counts are derived to ensure at least 80% power under these constraints.

Statistical analyses will be performed for dose escalation and both expansion parts of the study. All summaries will be reported by dose level, where applicable.

During part 1, the occurrence of dose-limiting toxicities and objective response rate will be assessed using a joint time-to-event continual reassessment model (26). Through use of this model, a recommended safe and active dose will be determined. For all parts, analysis will focus on AEs and preliminary activity. AEs will be graded according to the National Cancer Institute’s Common Terminology Criteria for Adverse Events version 5.0 and will be reported using the latest version of Medical Dictionary for Regulatory Activities coding dictionary. Objective response rate and disease control rate will be reported as percentages with 95% CI. Time-to-event endpoints will be described using Kaplan–Meier methods.

### Patient Engagement Strategy

Health authorities increasingly recognize the importance of patient-focused drug development to ensure that patients’ experiences, needs, and priorities are considered early in the clinical development process. Embedding patient centricity into trial design supports diverse recruitment and broader access to innovative treatments. It also enhances participants’ understanding, engagement, and retention, which ultimately benefits sponsors by improving study efficiency.

A key first step is engaging with patient advocacy groups early in trial design. Bayer’s Oncology Patient Council, including patients with advanced HCC, reviewed the BANTAM-01 study protocol and materials. Their feedback led to a more flexible schedule, centered on patient needs without compromising safety. Additional collaboration with the European Liver Patients’ Association and Liver Canada helped raise trial awareness, support recruitment, and provide participants with clear explanations of the study protocol.

Patient-facing elements were also integrated into the protocol through codesign with advisory groups. To ease logistical and financial burdens, which can be common barriers to trial participation, a patient concierge service was introduced, offering personalized travel support and reimbursements, particularly benefiting patients from lower socioeconomic backgrounds. To further improve understanding, the informed consent process was simplified with an eConsent system featuring summaries, audiovisual aids, and a glossary. Additional patient materials explain the trial’s goals and logistics in accessible terms, supporting participants throughout the trial. These materials are included in the supplemental materials for use by clinicians and advisory groups.

## DISCUSSION

Although several systemic therapies are available for the treatment of advanced HCC, response rates are suboptimal and 5-y survival remains low (8). These therapies are also associated with various toxicities, so there remains an unmet need for effective and well-tolerated targeted therapies. Targeting GPC3-expressing HCC tumors with ^225^Ac-GPC3 ACC is a promising alternative.

BANTAM-01’s dose escalation/expansion study design will define the safety profile, pharmacokinetics, and pharmacodynamics and evaluate preliminary antitumor activity of ^225^Ac-GPC3 ACC. The dose escalation part of the study will identify the recommended safe and active dose of ^225^Ac-GPC3 ACC, and the dose expansion parts will confirm the recommended dose and dosing regimen as monotherapy and in combination with SoC systemic therapies. Prospective screening for GPC3 expression ensures only participants who are good candidates to benefit from ^225^Ac-GPC3 ACC therapy enter the study.

Preclinical data support this first-in-human investigation of ^225^Ac-GPC3 ACC in advanced HCC, and the feasibility of targeting GPC3 has previously been shown or is currently being investigated in clinical trials with other GPC3-targeting agents. The TCE antibodies SAR444200 and ERY974 showed promising results in patients with advanced solid tumors; however, development for SAR444200 has been discontinued, and ERY974 is not actively being developed (18,27,28). Another TCE antibody, AZD9793, is currently in a phase 1/2 study for advanced or metastatic solid tumors (NCT06795022). GPC3-targeting CAR-T therapies under investigation include TAK-102 (NCT04405778), C-CAR031 (NCT06590246), and AZD5851 (NCT06084884). Additional agents targeting GPC3 in ongoing clinical trials for advanced HCC include RYZ801 and RYZ811 (NCT06726161), CM350 (the first GPC3/CD3 TCE bispecific antibody; NCT05263960), and CYT-303, a GPC3-targeting natural killer cell engager (29).

^225^Ac-labeled agents have also been investigated in other cancers. A phase 1 dose escalation study of ^225^Ac-J591, which targets prostate-specific membrane antigen, in patients with metastatic castration-resistant prostate cancer demonstrated reversible toxicity and antitumor activity (24). RYZ101, an ^225^Ac-labeled somatostatin receptor agonist, was well tolerated in a phase 1b trial (30), and a phase 3 study is currently ongoing. Furthermore, in preclinical studies, ^225^Ac-pelgifatamab (targeting prostate-specific membrane antigen) showed strong combination potential with the androgen receptor inhibitor, darolutamide, highlighting the potential of TAT in combination with established treatment regimens (31). A phase 1 study is ongoing (NCT06052306).

As previously highlighted, there is increased interest in patient-focused drug development in clinical trials and its importance in widening access to increase recruitment and encourage patient engagement and retention. A key focus of this study design, therefore, has been to maximize patient centricity through collaboration with patient advisory groups, gaining patient input on the study design, and providing patient-facing materials and other associated support programs. Although quantifying the impact of this approach on recruitment, retention, and overall study efficiency will be difficult, initial feedback from patient advisory groups has been overwhelmingly supportive. Any conclusions that can be drawn from this approach will be interesting to assess in the context of this study, while any useful learnings can potentially be applied to future clinical trials.

## CONCLUSION

There is an unmet need for effective and well-tolerated treatments in advanced HCC, and targeting GPC3-expressing HCC tumors with ^225^Ac-GPC3 ACC is a promising alternative to existing systemic therapies. ^225^Ac-GPC3 ACC is a potential first-in-class TAT in advanced HCC, and the BANTAM-01 study will provide important preliminary insights on its antitumor activity and safety profile.

## DISCLOSURE

This study is funded by Bayer AG. Arndt Vogel reports consultancy and advisory roles with Roche, AstraZeneca, Boehringer Ingelheim, Ipsen, Incyte, Cogent, Eisai, Zymeworks, Biologix, BMS, Terumo, Elevar, Servier, MSD, Tahio, Jazz Pharmaceuticals, Medivir, Abbvie, Tyra, Janssen, Lilly, and Bayer. Andrea Wilson Woods is employed by Blue Faery: The Adrienne Wilson Liver Cancer Association and has a consulting or advisory role with Eisai and Bayer. Daneng Li reports institutional grants or contracts from AstraZeneca and personal consulting fees from Jazz Pharmaceuticals, Bayer, AbbVie, TransThera, AstraZeneca, Coherus BioSciences, Eisai, Exelixis, Genentech, Merck, Trisalus Life Sciences, Sumitomo, Merus, Elevar Therapeutics, and Boehringer Ingelheim. Jorrit Tjeertes, Miroslav Dostalek, Charles Glaus, Vinita Gupta, Antje Nestler, Vasiliki Pelekanou, Joerg Pinkert, Ida Ratih, Eric Smith, Shyamal Subramanyam, Katrina Walker, Stefan Zimmermann, and Henrik Junger are employees of Bayer. No other potential conflict of interest relevant to this article was reported.

## Notes

KEY POINTS **QUESTION:** Is ^225^Ac-GPC3 ACC a safe and effective therapy for patients with GPC3-positive advanced HCC? **PERTINENT FINDINGS:** We describe the protocol and patient engagement strategy for the BANTAM-01 trial, a multicenter, open-label, first-in-human phase 1 dose escalation and expansion study of ^225^Ac-GPC3 ACC. Recruitment for the study began in March 2025. **IMPLICATIONS FOR PATIENT CARE:** The BANTAM-01 trial will generate data on the safety, tolerability, pharmacokinetics, and antitumor activity of ^225^Ac-GPC3 ACC, a potential first-in-class TAT, in patients with advanced HCC.
